# Adiposity Measurements and Metabolic Syndrome Are Linked Through Circulating Neuregulin 4 and Adipsin Levels in Obese Adults

**DOI:** 10.3389/fphys.2021.667330

**Published:** 2021-05-04

**Authors:** Dan Guo, Jianfang Liu, Peizhen Zhang, Xiaoyu Yang, Deying Liu, Jiayang Lin, Xueyun Wei, Bingyan Xu, Chensihan Huang, Xuan Zhou, Fei Teng, Hong Zhu, Huijie Zhang

**Affiliations:** ^1^Department of Endocrinology and Metabolism, Nanfang Hospital, Southern Medical University, Guangzhou, China; ^2^Department of Endocrinology and Diabetes, The First Affiliated Hospital, Xiamen University, Xiamen, China; ^3^Nanfang Hospital, Southern Medical University, Guangzhou, China; ^4^School of Public Health, Southern Medical University, Guangzhou, China

**Keywords:** neuregulin 4, adipsin, adiposity, metabolic syndrome, metabolism, mediation analysis

## Abstract

**Background:**

Adiposity and adipokines are associated with metabolic disorders, but little is known regarding that whether adiposity measurements link metabolic syndrome (MetS) through circulating neuregulin 4 (Nrg4) and adipsin levels.

**Materials and Methods:**

A total of 1212 subjects with a waist circumference greater than 90 cm for men or 80 cm for women were enrolled from a Chinese community. Circulating Nrg4 and adipsin levels were measured using commercial kits. Mediation analyses of circulating Nrg4 and adipsin were performed in the study using linear and logistic regression.

**Results:**

Subjects with MetS had higher waist circumference, visceral fat level, and circulating adipsin level, and lower levels of circulating Nrg4 and muscle mass to visceral fat (MVF) ratio (all *P* < 0.05). In multivariable logistic regression analyses, after adjusting for confounding variables, per standard deviation (SD) increase in waist circumference and visceral fat level were significantly associated with increased odds of MetS [OR (95% CI), 1.42 (1.22–1.64); 2.20 (1.62–2.99); respectively]; and per SD reduction in MVF ratio was significantly associated with reduced odds of MetS [OR (95% CI), 0.65 (0.55–0.77)]. In the mediation analyses, both circulating Nrg4 and adipsin levels mediated the association between waist circumference (8.31% and 18.35%, respectively), visceral fat level (7.50% and 9.98%, respectively), and MVF ratio (5.80% and 9.86%, respectively) and MetS after adjustments.

**Conclusion:**

These findings indicate that adiposity measurements and MetS are linked through circulating Nrg4 and adipsin levels in obese adults, suggesting that circulating Nrg4 and adipsin levels might be potential predictors for management of MetS.

## Introduction

Metabolic syndrome (MetS) is a constellation of medical conditions, including central obesity, hypertension, hyperglycemia, and dyslipidemia, which are associated with increased risk of cardiometabolic diseases such as cardiovascular diseases (CVD) and type 2 diabetes (Lorenzo et al., 2003; Alberti et al., 2005; Park et al., 2019). MetS has an increasing prevalence worldwide owing to energy-dense dietary intake, sedentary lifestyles, and consequently excess visceral fat accumulation (Cameron et al., 2008; Hirode and Wong, 2020). Although the pathogenesis of MetS has not been fully elucidated, low-grade inflammation induced by central obesity through adipokine dysregulation has been established as one of the significant underlying mechanisms of MetS (Saltiel and Olefsky, 2017; Francisco et al., 2019).

Adipose tissue is recognized as not only an energy storage site but also an endocrine organ secreting a series of adipokines, which are identified to play important roles in metabolic disorders and CVD (Kahn et al., 2019; Sanidas et al., 2020). For instance, it has been documented that Neuregulin 4 (Nrg4) primarily secreted from brown adipose tissue (BAT) and adipsin mainly synthesized in the white adipose tissue (WAT) are associated with metabolic diseases (Saleh et al., 2019; Tutunchi et al., 2020). Nrg4 is a newly identified adipokine and play a role in obesity, diabetes, non-alcoholic fatty liver disease (NAFLD), and CVD in human subjects (Wang et al., 2014; Dai et al., 2015; Jiang et al., 2016; Chen et al., 2017; Yan et al., 2019). Moreover, our previous study found that serum Nrg4 levels were negatively associated with MetS (Cai et al., 2016). The key mechanistic link for Nrg4 in protecting from metabolic disturbances is to regulate BAT activity, drive the browning of WAT, prevent lipogenesis in the liver, and improve insulin sensitivity (Tutunchi et al., 2020). Adipsin has been documented to regulate adipocyte differentiation and increased lipid accumulation, which might be a potential reason for the association between adipsin and metabolic disorders (Song et al., 2016). Clinical evidences indicated that circulating adipsin were positively associated with adiposity, polycystic ovary syndrome (PCOS), insulin resistance (IR), and the development of coronary artery disease (Derosa et al., 2013; Gursoy Calan et al., 2016; Guo et al., 2019; Ohtsuki et al., 2019).

As mentioned above, both Nrg4 and adipsin secreted from adipose tissue have impacts on metabolic abnormalities. However, whether adiposity measurements link MetS through Nrg4 and adipsin remains unknown in humans. In this study, we aimed to explore whether the associations of adiposity measurements with MetS are mediated by circulating Nrg4 and adipsin levels and further quantify the degree of the mediation effects in obese subjects.

## Subjects and Methods

### Study Participants

Obese subjects living in the Lianqian community, Xiamen, China were recruited between April 2011 and December 2013. Adults with central obesity (waist circumference ≥90 cm for men or 80 cm for women) were eligible if they were aged 18 or older. Subjects with viral hepatitis, liver cirrhosis, any secondary chronic liver diseases (e.g., alcoholic fatty liver, autoimmune hepatitis, and/or drug-induced hepatitis), biliary obstructive diseases, cancer, or known hyper- or hypothyroidism were excluded. In brief, a total of 1529 obese subjects were included, of which 1212 (79.3%) participants with complete data were left for further analysis. All participants completed a physical examination and a uniform questionnaire covered social demographic status, family history, past medical history, and lifestyle habits such as smoking status, alcohol consumption, and physical activity. Written informed consent was provided by participants. The methods were carried out in accordance with the approved guidelines.

### Clinical and Biochemical Measurements

Basic anthropometric measurements used in the analysis included height, weight, waist circumference, blood pressure (BP). Body mass index (BMI) was calculated as the weight in kilograms divided by the square of the height in meters. Waist circumference was measured at the level of the umbilicus. Each measurement was repeated for three times, and the results were averaged. BP was assessed in triplicate after at least 5 minutes of rest using an electronic sphygmomanometer (OMRON Company), and the mean values were used for analysis. Visceral fat level and muscle mass were determined using the whole body dual-energy X-ray absorptiometry (DXA) system (Hologic Inc., Bedford, MA, United States). Muscle mass to visceral fat (MVF) ratio was calculated as the muscle mass divided by visceral fat level.

All blood samples were obtained after 12 h of fasting or 2 h after 75-g oral glucose tolerance test which was performed to assess glucose tolerance status. Fasting plasma glucose concentrations (FPG) and 2-h plasma glucose concentrations (2-h PG) were determined using the glucose oxidase method. Fasting plasma insulin concentrations and 2-h plasma insulin concentrations were determined using an electrochemiluminescence immunoassay (Roche Elecsys Insulin Test, Roche Diagnostics, Mannheim, Germany). Serum lipid profiles, including triglycerides (TG), total cholesterol (TC), and high-density lipoprotein cholesterol (HDL-c) were measured by enzymatic colorimetric methods with a Hitachi 7450 analyzer (Hitachi, Tokyo, Japan). Low-density lipoprotein cholesterol (LDL-c) was calculated by Friedewald’s formula. The homeostasis model assessment of insulin resistance (HOMA-IR) was calculated as the product of fasting serum insulin (mU/L) and FPG (mmol/L) concentrations, divided by 22.5.

### Measurements of Circulating Nrg4 and Adipsin Concentrations

Circulating Nrg4 concentrations were measured using enzyme-linked immunosorbent assay (ELISA) kits (Aviscera Biosciences, Santa Clara, CA, United States), following the manufacturer’s protocol. The assay had a detection sensitivity of 0.25 ng/mL. The linear range of the standard was 0.5–32.0 ng/mL, and the intra- and inter-assay variations were both less than 10%.

Circulating adipsin concentrations were assayed by using ELISA kits (AssayPro, St. Charles, MO, United States). The assay had a detection sensitivity of 2.1 ng/mL. The linear range of the standard was 1.875–120 ng/mL, and the intra- and inter-assay variations were both less than 10%.

### Definition of Metabolic Syndrome

MetS was defined using the criteria proposed by International Diabetes Federation diagnostic criteria (Alberti et al., 2005), which consisted of central obesity (waist circumference ≥90 cm for Chinese men or ≥80 cm for Chinese women) and the presence of any two of four risk factors: (1) elevated BP (≥130/85 mmHg or drug treatment of previously diagnosed hypertension), (2) elevated fasting plasma glucose (≥5.6 mmol/L or previously diagnosed type 2 diabetes), (3) elevated triglyceride (≥1.7 mmol/L, or drug treatment for this lipid abnormality), or (4) reduced HDL-c (<1.03 mmol/L for men or <1.29 mmol/L for women, or drug treatment for this lipid abnormality). The latter two risk factors, elevated triglyceride level and reduced HDL-c, were collectively referred to as dyslipidemia in the present study.

### Statistical Analysis

Statistical analyses were performed with SAS version 9.4 (SAS Institute, Cary, NC, United States). All data are presented as proportions for categorical variables or means ± standard deviations (SD) for continuous variables, except for skewed variables, which are presented as medians (interquartile range). Data that were not normally distributed were logarithmically transformed prior to analysis. The subjects were classified into two groups according to different metabolic status. The χ2-test was used for comparison of categorical variables between groups. Analyses of covariance were performed using general linear models (GLM) to compare the differences in study variables between groups. For adiposity measurements, circulating Nrg4 and adipsin, standardized values (Z-scores) were calculated to allow direct comparison of their effect sizes in the regression analyses. Logistic regression analyses were used to examine the association between adiposity measurements and MetS or components of MetS. The results of the analyses are presented as odds ratio (OR) with a 95% confidence interval (CI).

General causal mediation analysis models previously proposed by VanderWeele and Sobel (Sobel, 1982; VanderWeele, 2016) were constructed to explore whether Nrg4 or adipsin mediated the effect of adiposity measurements on MetS. Linear and logistic regression models were used to estimate standardized regression coefficients (βs). In the mediation models, the predictor variables (X) were waist circumstance, visceral fat level, or MVF ratio; the mediator variables (M) were Nrg4 or adipsin; the outcome variable (Y) was MetS. The mediation model is presented in Supplementary Figure 1. Four steps of the mediation analysis were involved in the calculation of the mediating effect:

Step 1: Showing that the predictor variable determines the outcome (Model Y = β_*Tot*_X) [β_*Tot*_ is the coefficient relating the predictor variable to the outcome variable (total effect)].

Step 2: Showing that the predictor variable affects the mediator (Model M = β_1_X) [β_1_ is the coefficient relating the predictor variable to the mediator variable (indirect effect 1)].

Step 3: Showing that the mediator determines the outcome controlling for the predictor (Model Y = β_2_M + β_*Dir*_X) [β_2_ is the coefficient relating the mediator variable to the outcome variable adjusted for the predictor variable (indirect effect 2); β_*Dir*_ is the coefficient relating the predictor variable to the outcome variable adjusted for the mediator variable (direct effect)].

Step 4: Calculating the proportion of mediation effect (%) by (β_1_ × β_2_/β_*Tot*_) × 100%.

The total indirect effect (β_*Ind*_) was calculated by multiplying the regression coefficients β_1_ × β_2._ In mediation analyses, testing the significance of the mediation effect is equivalent to testing the null hypothesis H_0_: β_1_ × β_2_ = 0 versus the alternative hypothesis H_*a*_: β_1_ × β_2_≠0, by using the Sobel test. Two-sided values of *P* < 0.05 were considered statistically significant.

## Results

Table 1 summarizes the mean levels of study variables according to different components of MetS. Of the 1212 eligible obese subjects, 781 (64.4%) had the MetS, 707 (58.3%) had elevated BP, 715 (60.0%) had elevated fasting glucose, and 704 (58.1%) had dyslipidemia. Female and older people are more likely to have MetS and elevated BP. As expected, subjects with each components of metabolic disorders had an adverse metabolic profile, including significantly higher levels of BMI, waist circumference, visceral fat level, systolic and diastolic BP, fasting plasma glucose and insulin, postprandial glucose and insulin, triglyceride, total cholesterol, and HOMA-IR, and lower levels of HDL-c and MVF ratio. The circulating adipsin level was markedly higher in subjects with MetS (*P* = 0.019) and elevated BP (*P* = 0.001); meanwhile, it was not significantly associated with elevated fasting glucose and dyslipidemia. The circulating Nrg4 level was significantly lower in subjects with MetS (*P* < 0.001), elevated BP (*P* = 0.010), elevated fasting glucose (*P* = 0.002), and dyslipidemia (*P* = 0.034). Furthermore, female subjects with MetS had a higher level of circulating adipsin and a lower level of circulating Nrg4, while the difference of these two adipokines were not statistically significant in male subjects between groups (Supplementary Table 1).

**TABLE 1:** Characteristics of obese subjects according to components of metabolic syndrome.

Variables	Metabolic syndrome			Elevated blood pressure			Elevated Fasting Glucose			Dyslipidemia		
				
	Yes	No	*P*-value	Yes	No	*P*-value	Yes	No	*P*-value	Yes	No	*P*-value
Sample size	781	431		707	505		715	497		704	508	
Age (years)	54.3 ± 7.2	51.6 ± 7.2	<0.001	54.7 ± 7.0	51.4 ± 7.2	<0.001	54.6 ± 6.9	51.5 ± 7.4	<0.001	53.5 ± 7.3	53.2 ± 7.3	0.496
Female (n,%)	530 (67.9)	335 (77.7)	<0.001	457 (64.6)	408 (80.8)	<0.001	509 (71.2)	356 (71.6)	0.867	480 (68.1)	385 (75.8)	0.004
BMI (kg/m^2^)	27.8 ± 3.2	26.8 ± 2.7	<0.001	27.9 ± 3.2	26.8 ± 2.8	<0.001	27.7 ± 3.2	27.1 ± 2.8	0.003	27.7 ± 3.0	27.1 ± 3.1	<0.001
Waist circumference (cm)	94.7 ± 7.4	92.2 ± 6.6	<0.001	95.0 ± 7.7	92.1 ± 6.2	<0.001	94.4 ± 7.4	93.0 ± 7.0	0.002	94.3 ± 7.0	93.1 ± 7.5	0.002
Current smokers (n, %)	104 (13.3)	54 (12.5)	0.697	110 (15.6)	48 (9.5)	0.002	94 (13.2)	64 (12.9)	0.891	87 (12.4)	71 (14.0)	0.409
Systolic BP (mmHg)	139.2 ± 16.4	122.6 ± 14.2	<0.001	144.1 ± 14.4	118.2 ± 7.6	<0.001	136.4 ± 17.2	128.9 ± 17.1	<0.001	135.5 ± 17.1	130.3 ± 17.7	<0.001
Diastolic BP (mmHg)	82.3 ± 10.3	73.7 ± 8.7	<0.001	84.7 ± 9.7	71.7 ± 6.4	<0.001	80.6 ± 10.4	77.3 ± 10.6	<0.001	80.7 ± 10.4	77.2 ± 10.6	<0.001
Fasting glucose (mmol/L)	6.49 ± 1.91	5.49 ± 0.87	<0.001	6.37 ± 1.93	5.80 ± 1.18	<0.001	6.76 ± 1.95	5.23 ± 0.26	<0.001	6.34 ± 1.97	5.85 ± 1.13	<0.001
2-h glucose (mmol/L)	9.91 ± 4.38	7.23 ± 2.28	<0.001	9.60 ± 4.30	8.05 ± 3.28	<0.001	10.23 ± 4.56	7.12 ± 1.75	<0.001	9.66 ± 4.48	7.97 ± 2.90	<0.001
Fasting insulin (mU/L)	14.86 ± 13.64	10.05 ± 4.64	<0.001	14.21 ± 14.01	11.68 ± 6.37	<0.001	14.68 ± 14.09	10.96 ± 5.53	<0.001	14.63 ± 14.02	11.11 ± 6.14	<0.001
2-h insulin (mU/L)	94.43 ± 71.21	61.70 ± 46.69	<0.001	89.50 ± 70.52	73.41 ± 56.40	<0.001	91.70 ± 72.73	69.98 ± 50.71	<0.001	92.57 ± 70.46	69.25 ± 55.11	<0.001
HOMA-IR	3.47 (2.54–4.89)	2.19 (1.56–2.99)	<0.001	3.23 (2.33–4.63)	2.60 (1.77–3.89)	<0.001	3.54 (2.59–5.11)	2.28 (1.60–3.10)	<0.001	3.33 (2.40–4.74)	2.54 (1.74–3.53)	<0.001
Triglyceride (mmol/L)	1.97 (1.37–2.67)	1.13 (0.85–1.41)	<0.001	1.78 (1.18–2.52)	1.33 (0.93–1.96)	<0.001	1.66 (1.16–2.39)	1.40 (0.95–2.03)	<0.001	2.12 (1.72–2.88)	1.09 (0.85–1.34)	<0.001
Total cholesterol (mmol/L)	6.01 ± 1.13	5.70 ± 0.97	<0.001	6.03 ± 1.13	5.71 ± 0.99	<0.001	6.04 ± 1.11	5.70 ± 1.02	<0.001	5.97 ± 1.18	5.81 ± 0.94	<0.001
LDL-c (mmol/L)	3.68 ± 1.04	3.65 ± 0.90	0.533	3.74 ± 1.04	3.57 ± 0.91	0.003	3.76 ± 1.03	3.55 ± 0.92	<0.001	3.60 ± 1.06	3.77 ± 0.89	0.004
HDL-c (mmol/L)	1.29 ± 0.27	1.51 ± 0.28	<0.001	1.34 ± 0.29	1.40 ± 0.29	0.001	1.35 ± 0.29	1.39 ± 0.30	0.020	1.23 ± 0.22	1.56 ± 0.27	<0.001
Adipsin (μg/ml)	5.02 (4.05–6.34)	4.84 (3.93–6.16)	0.019	5.13 (4.05–6.44)	4.79 (3.97–6.03)	0.001	4.96 (3.97–6.18)	4.90 (4.06–6.34)	0.794	4.98 (4.05–6.31)	4.90 (3.93–6.23)	0.149
Nrg4 (ng/ml)	3.24 (2.40–4.52)	3.55 (2.60–5.29)	<0.001	3.26 (2.37–4.59)	3.47 (2.60–5.05)	0.010	3.21 (2.38–4.58)	3.53 (2.60–5.05)	0.002	3.32 (2.50–4.65)	3.41 (2.41–5.02)	0.034
Visceral fat level	10 (8–14)	8 (7–11)	<0.001	10 (8–15)	8 (7–10.5)	<0.001	9 (8–14)	9 (8–13)	0.013	9 (8–14)	9 (7–12)	<0.001
MVF ratio	4.22 ± 0.80	4.64 ± 0.93	<0.001	4.17 ± 0.79	4.66 ± 0.90	<0.001	4.28 ± 0.85	4.51 ± 0.89	<0.001	4.28 ± 0.83	4.51 ± 0.92	<0.001

*Data are presented as the mean ± SD or median (interquartile range).*

*BMI, body mass index; BP, blood pressure; HOMA-IR, homeostasis model assessment of insulin resistance; LDL-c, low-density lipoprotein cholesterol; HDL-c, high -density lipoprotein cholesterol; MVF, muscle mass to visceral fat; Nrg4, neuregulin 4.*

Table 2 presents the total effect of adiposity measurements on different components of MetS. Waist circumstance, visceral fat level, and MVF ratio were significantly associated with odds of metabolic components. The ORs for the association of waist circumstance with MetS, elevated BP, elevated fasting glucose, and dyslipidemia were 1.48 (95% CI, 1.29–1.69), 1.58 (95% CI, 1.39–1.80), 1.21 (95% CI, 1.08–1.37), and 1.20 (95% CI, 1.07–1.35), respectively. Furthermore, the adiposity measurements were significantly associated with MetS and elevated BP in both male and female subjects (Supplementary Figure 2). The associations between adiposity measurements and different components of MetS remained significant after additional adjustments of age, gender, smoking status, alcohol assumption, and physical activity, and even further adjustments of total cholesterol and LDL-c. Of note, per SD increase of visceral fat level was markedly associated with the increased odds of MetS [OR (95% CI), 2.20 (1.62–2.99); *P* < 0.001], elevated BP [OR 95% CI), 2.04 (1.52–2.75); *P* < 0.001], elevated fasting glucose [OR (95% CI), 1.50 (1.13–1.98); *P* < 0.01], and dyslipidemia [OR (95% CI), 1.75 (1.31–2.34); *P* < 0.01] in the final model.

**TABLE 2:** Effect of adiposity measurements on the components of metabolic syndrome.

	Metabolic syndrome	Elevated blood pressure	Elevated fasting glucose	Dyslipidemia
				
	OR (95%CI)	OR (95%CI)	OR (95%CI)	OR (95%CI)
**Model 1**				
Waist circumference	1.48 (1.29–1.69)***	1.58 (1.39–1.80)***	1.21 (1.08–1.37)**	1.20 (1.07–1.35)**
Visceral fat level	1.50 (1.32–1.71)***	1.74 (1.52–1.98)***	1.16 (1.03–1.31)*	1.29 (1.14–1.45)***
MVF ratio	0.61 (0.54–0.69)***	0.55 (0.48–0.63)***	0.76 (0.68–0.86)***	0.76 (0.68–0.86)***
**Model 2**				
Waist circumference	1.39 (1.21–1.61)***	1.40 (1.22–1.61)***	1.22 (1.07–1.39)**	1.15 (1.01–1.30)*
Visceral fat level	1.99 (1.48–2.68)***	1.94 (1.45–2.60)***	1.44 (1.09–1.90)*	1.56 (1.18–2.06)**
MVF ratio	0.66 (0.56–0.78)***	0.68 (0.58–0.81)***	0.81 (0.69–0.95)*	0.76 (0.65–0.89)***
**Model 3**				
Waist circumference	1.42 (1.22–1.64)***	1.41 (1.23–1.62)***	1.22 (1.07–1.39)**	1.18 (1.03–1.34)*
Visceral fat level	2.20 (1.62–2.99)***	2.04 (1.52–2.75)***	1.50 (1.13–1.98)**	1.75 (1.31–2.34)**
MVF ratio	0.65 (0.55–0.77)***	0.68 (0.57–0.80)***	0.81 (0.69–0.96)*	0.73 (0.62–0.87)***

OR, odds ratio; CI, confidence interval; MVF, muscle mass to visceral fat.

*P < 0.05; **P < 0.01; ***P < 0.001.

*Model 1: without adjustment. Model 2: adjusted for age, gender, smoking, alcohol assumption, and physical activity. Model 3: adjusted for model 2 + total cholesterol, LDL- cholesterol.*

**FIGURE 1:**
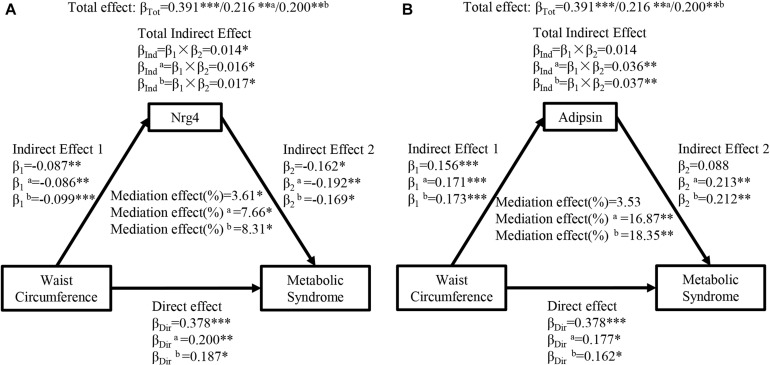
Ngr 4 and adipsin mediate the effect of waist circumference on metabolic syndrome. **(A)** Mediation analysis of Nrg4. **(B)** Mediation analysis of adipsin. β, standardized regression coefficient; β_1_, indirect effect 1; β_2_, indirect effect 2; β_*Ind*_, total indirect effect; β_*Dir*_, direct effect; β_*Tot*_, total effect; Nrg4, neuregulin 4. *P < 0.05; **P < 0.01; ***P < 0.001. Model a: adjusted for fasting insulin, 2-h glucose. Model b: adjusted for model a + HOMA-IR.

Figure 1 shows the mediation effects of circulating Nrg4 and adipsin levels on the association between waist circumference and MetS. The total effect of waist circumference on MetS was measured as a standardized regression coefficient (β_*Tot*_ = 0.391, *P* < 0.001). The waist circumference was inversely correlated with circulating Nrg4 and was positively correlated with circulating adipsin (β_1_ = −0.087, *P* < 0.01; β_1_ = 0.156, *P* < 0.001, respectively). The total indirect effect (β_*Ind*_ = 0.014, *P* < 0.05) through circulating Nrg4 level defined as the product of indirect effect 1 (β_1_) and indirect effect 2 (β_2_) was significant (Figure 1A). The mediation effect of circulating Nrg4 level on the waist circumference-MetS association was 3.61%. After adjusting for fasting insulin and postprandial glucose (Figure 1B, model a), the mediation effect of circulating adipsin and Nrg4 levels on the waist circumference-MetS association was estimated at 16.87%, 7.66%, respectively. Both the mediation effects were increased from 7.66% to 8.31% for circulating Nrg4 and from 16.87% to 18.35% for circulating adipsin with further adjustment of HOMA-IR (Model b).

**FIGURE 2:**
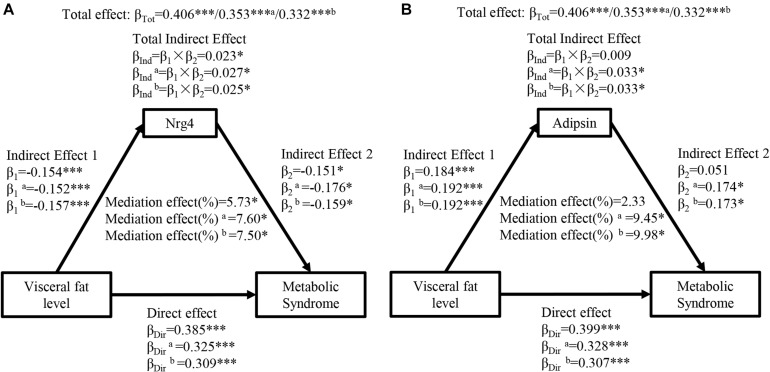
Ngr 4 and adipsin mediate the effect of visceral fat level on metabolic syndrome. **(A)** Mediation analysis of Nrg4. **(B)** Mediation analysis of adipsin. β, standardized regression coefficient; β_1_, indirect effect 1; β_2_, indirect effect 2; β_*Ind*_, total indirect effect; β_*Dir*_, direct effect; β_*Tot*_, total effect; Nrg4, neuregulin 4. Model a: adjusted for fasting insulin, 2-h glucose. Model b: adjusted for model a + HOMA-IR. *P < 0.05; ***P < 0.001.

Figure 2 presents the mediation effects of circulating Nrg4 and adipsin levels on the association between visceral fat level and MetS. The total effect of visceral fat level on MetS was notable (β_*Tot*_ = 0.406, *P* < 0.001). The visceral fat level had a negative correlation with circulating Nrg4 and a positive correlation with circulating adipsin (β_1_ = −0.154, *P* < 0.001; β_1_ = 0.184, *P* < 0.001, respectively). Circulating Nrg4 had a significant mediation effect on the association of visceral fat level and MetS (percent mediated effect, 5.73%), and the mediation effect increased to 7.60% and 7.50% in model a and model b, respectively (Figure 2A). After adjusting for several confounding factors, visceral fat level was linked to MetS through circulating adipsin with a mediation effect of 9.45% in model a and a mediation effect of 9.98% in model b (Figure 2B).

**FIGURE 3:**
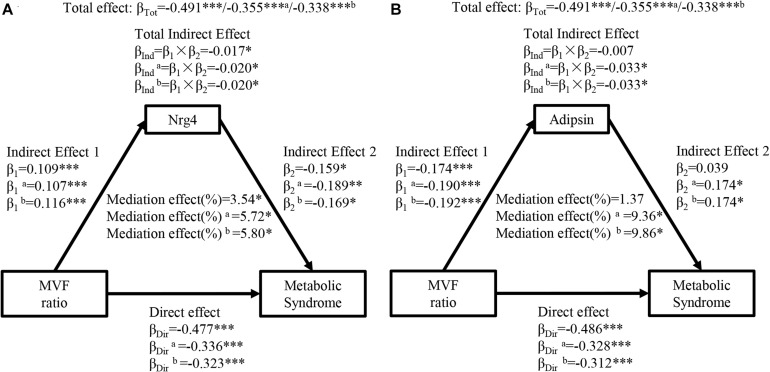
Ngr 4 and adipsin mediate the effect of MVF ratio on metabolic syndrome. **(A)** Mediation analysis of Nrg4. **(B)** Mediation analysis of adipsin. β, standardized regression coefficient; β_1_, indirect effect 1; β_2_, indirect effect 2; β_*Ind*_, total indirect effect; β_*Dir*_, direct effect; β_*Tot*_, total effect; MVF, muscle mass to visceral fat; Nrg4, neuregulin 4. Model a: adjusted for fasting insulin, 2-h glucose. Model b: adjusted for model a + HOMA-IR. *P < 0.05; **P < 0.01; ***P < 0.001.

Figure 3 shows the mediation effects of circulating Nrg4 and adipsin levels on the association between MVF ratio and MetS. The MVF ratio had a significant effect on MetS (β_*Tot*_ = 0.491, *P* < 0.001). The MVF ratio was positively correlated with circulating Nrg4 and negatively correlated with circulating adipsin (β_1_ = 0.109, *P* < 0.001; β_1_ = −0.174, *P* < 0.001; respectively). Similarly, circulating Nrg4 also partially mediated the effect of MVF ratio on MetS with a mediation effect of 3.54%, which was elevated to 5.72% and 5.80% with further adjustments (Figure 3A). A significant mediation effect of circulating adipsin was presented after adjusting the confounders (percent mediated effect, 9.36% and 9.86% respectively in two models, Figure 3B). In addition, the interaction effects of circulating Nrg4 and adipsin by gender on the association between adiposity measurements and MetS were not significant (all *P* > 0.05; Supplementary Figures 3, 4, respectively).

## Discussion

As an endocrine organ, adipose tissue regulates metabolic homeostasis by releasing a large number of adipokines (Kahn et al., 2019). Recently, several novel proteins secreted from adipocytes, such as Nrg4 and adipsin have been identified to play a role in modulate systemic metabolic homeostasis and are associated with MetS and cardiovascular disease (Saleh et al., 2019; Tutunchi et al., 2020). However, it was unclear whether and to what extent adiposity affects MetS through circulating Nrg4 and adipsin levels in humans. In the present study, our findings highlighted that adiposity measurements, including waist circumference, visceral fat level, and MVF ratio, were significantly associated with MetS through circulating Nrg4 and adipsin levels. This study provides strong evidence that the effect of adiposity on the development of MetS is mediated by secretory adipokines.

It has been generally accepted that adiposity is associated with MetS. Several studies have indicated waist circumference, visceral fat level, and MVF ratio were useful measurements for the detection of MetS (Unno et al., 2012; Yu et al., 2016; Ramírez-Vélez et al., 2018). Consistently, we found that waist circumference and visceral fat level were significantly associated with increased odds of MetS and its each component. Besides, our study also found that increased MVF ratio was associated with lower odds of MetS.

It has been established that adiposity is inversely correlated to BAT mass or activity in adult human, which protects against obesity and related metabolic disorders by promoting energy expenditure via the activity of uncoupling protein 1 (UCP1) (Kahn et al., 2019). Nrg4 is identified a brown-fat-enriched endocrine factor with therapeutic potential for the treatment of obesity-associated disorders (Tutunchi et al., 2020). Evidence has suggested that Nrg4 was inversely correlated with adiposity (Jiang et al., 2016; Chen et al., 2017; Yan et al., 2019) and might be a protective factor in the development of MetS (Cai et al., 2016). However, it was unclear to whether adiposity affects MetS through circulating Nrg4 level. Our results showed that adiposity measurements were significantly associated with MetS through decreased circulating Nrg4 level. Furthermore, we quantified the mediation effect of circulating Nrg4 level in the association of waist circumference, visceral fat level, and MVF ratio with MetS was 8.31%, 7.50%, and 5.80%, respectively. These observations provided additional, stronger evidence to support previous findings from our (Cai et al., 2016) and other studies (Wang et al., 2014; Dai et al., 2015; Jiang et al., 2016; Chen et al., 2017; Yan et al., 2019) that brown fat-enriched secreted factor Nrg4 is involve in crosstalk between adiposity and metabolic disorders.

Adiposity is characterized by a substantial increase in WAT mass and is associated with increased risk of MetS. Adipsin mainly synthesized and secreted from WAT, plays a role in modulating lipid and glucose metabolism (Lo et al., 2014; Gómez-Banoy et al., 2019). Several clinical studies reported that elevated serum adipsin levels was observed in obese subjects and positively associated with BMI and visceral adipose tissue (Abu-Farha et al., 2014; Gursoy Calan et al., 2016; Schrover et al., 2018; Ohtsuki et al., 2019). Schrover et al. (2018) reported that adipsin was not significant associated with MetS in patients with clinically manifest vascular disease. Consistently, our data indicated that circulating adipsin was positively correlated with waist circumference and visceral fat level, and negatively associated with MVF ratio. Besides, our results also demonstrated that subjects with MetS had a higher level of circulating adipsin. Of note, our mediation analyses showed that increased adiposity had an adverse effect on MetS which was partially mediated through circulating adipsin levels, with a mediation effect of 18.35% accounting for waist circumference-MetS association and 9.98% accounting for visceral fat level-MetS association. The mediation effect might be explained by the function of adipsin, which increases lipid accumulation and adipocyte differentiation through inducing the peroxisome proliferator-activated receptor γ (Pparγ) and releasing complement component 3a (C3a) (Song et al., 2016; Saleh et al., 2019). Our data suggests that the expansion of WAT increased the level of circulating adipsin which might have a potential impact on the development of MetS.

Of note, the mediation effects of circulating Nrg4 and adipsin remained significant after adjusting several variables related to IR, suggesting that the potential mechanism of circulating Nrg4 and adipsin mediating the association between adiposity and MetS is independent of IR. Similarly, recent study carried out in the Netherlands also reported that the association between abdominal adiposity and HOMA-IR was mediated by leptin and adiponectin (Noordam et al., 2020). These observations provided stronger evidence that adipokines secreted by adipocytes is involved in crosstalk between adiposity and metabolic disorders. However, the underlying mechanism of different adipokines in the pathogenesis of obesity-induced MetS needs to be further studied in *vivo* and in *vitro*.

The novelty of this community-based, cross-sectional study is that we examined and quantified the degree of the mediation effect of circulating Nrg4 and adipsin on the association between adiposity and MetS. We found that the path from adiposity and MetS was protectively mediated Nrg4 but adversely mediated by adipsin, suggesting that elevating circulating Nrg4 levels and inhibiting adipsin secreting might be therapeutic potentials for protecting obesity from MetS. These findings indicate that adipokines could be used as predictors of metabolic phenotype and monitoring markers for lifestyle intervention and weight management in the future. However, there are several limitations to the current study. First, given its cross-sectional design, we cannot exclude the pathways of associations may exist an inverse direction. Second, the visceral fat in the study was measured using DXA which is not a gold standard tool. However, studies have demonstrated that DXA is comparable with computed tomography (CT) and magnetic resonance imaging (MRI) with R^2^ ranging between 0.82 to 0.96, as a result, DXA is considered as a feasible alternative (Browning et al., 2011; Neeland et al., 2018, 2019). Third, our study population focus on the Chinese adults and therefore the findings may not be generalizable to other ethnicities or geographic regions. Therefore, the pathways of the association should be further evaluated in prospective studies with larger sample sizes, different ethnicities, and long follow-up periods.

## Conclusion

Our study indicated that the association between adiposity measurements and MetS was linked through circulating Nrg4 and adipsin levels. The quantification of the mediation effects of circulating Nrg4 and adipsin levels may facilitate the development of novel prevention and intervention strategies for controlling the progression from adiposity to MetS.

## Data Availability Statement

The raw data supporting the conclusions of this article will be made available by the authors, without undue reservation.

## Ethics Statement

The studies involving human participants were reviewed and approved by the Institutional Review Board of The First Affiliated Hospital of Xiamen University. The patients/participants provided their written informed consent to participate in this study.

## Author Contributions

All authors listed have made a substantial, direct and intellectual contribution to the work, and approved it for publication.

## Conflict of Interest

The authors declare that the research was conducted in the absence of any commercial or financial relationships that could be construed as a potential conflict of interest.
