# Supplementary figures and images for: Adiposity Measurements and Metabolic Syndrome Are Linked Through Circulating Neuregulin 4 and Adipsin Levels in Obese Adults
Source: Front Physiol. 2021 May 4;12:667330. doi: 10.3389/fphys.2021.667330 (PMC8129583; doi:10.3389/fphys.2021.667330)

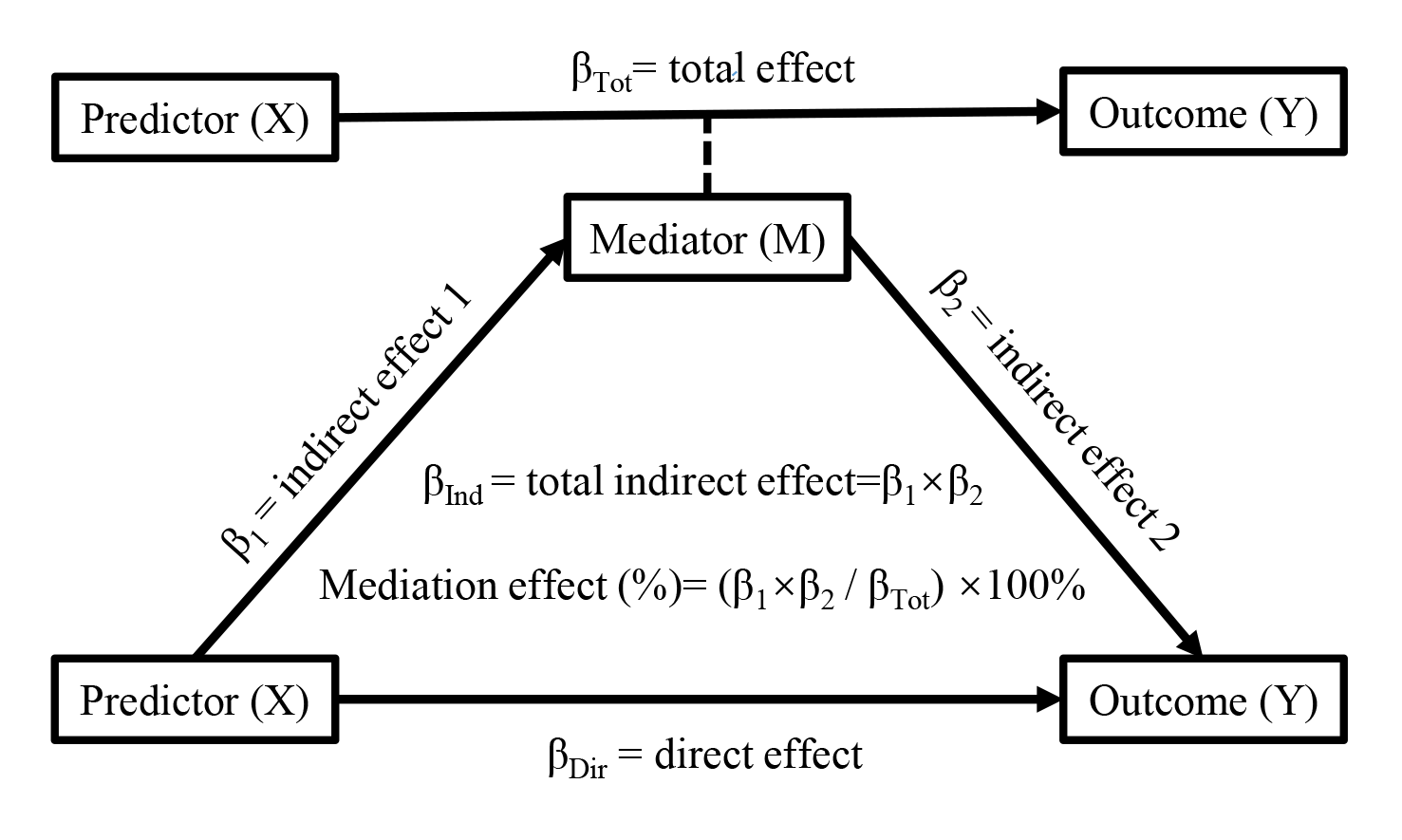

Supplement: Supplementary file 1 [file Image_1.TIF]

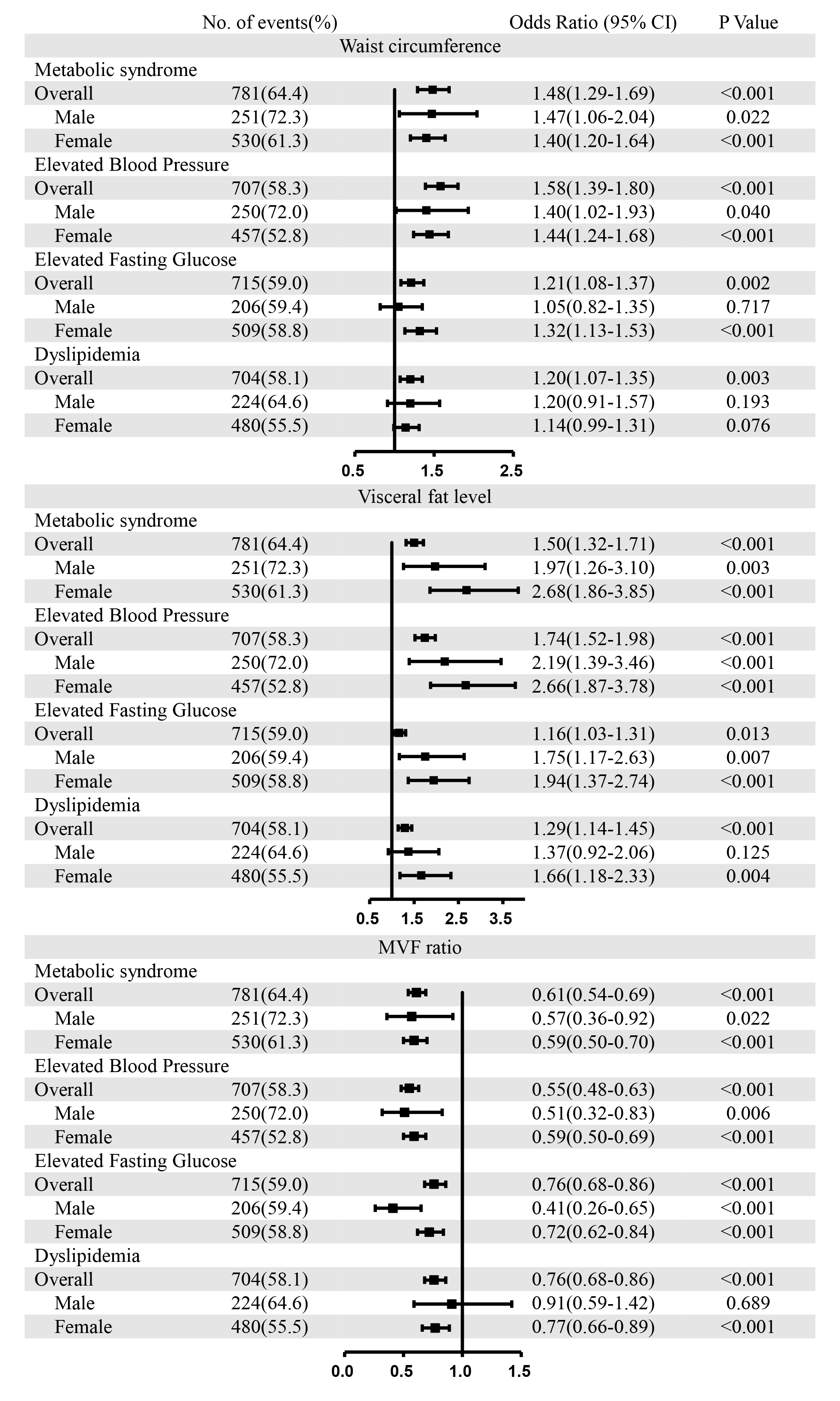

Supplement: Supplementary file 2 [file Image_2.TIF]

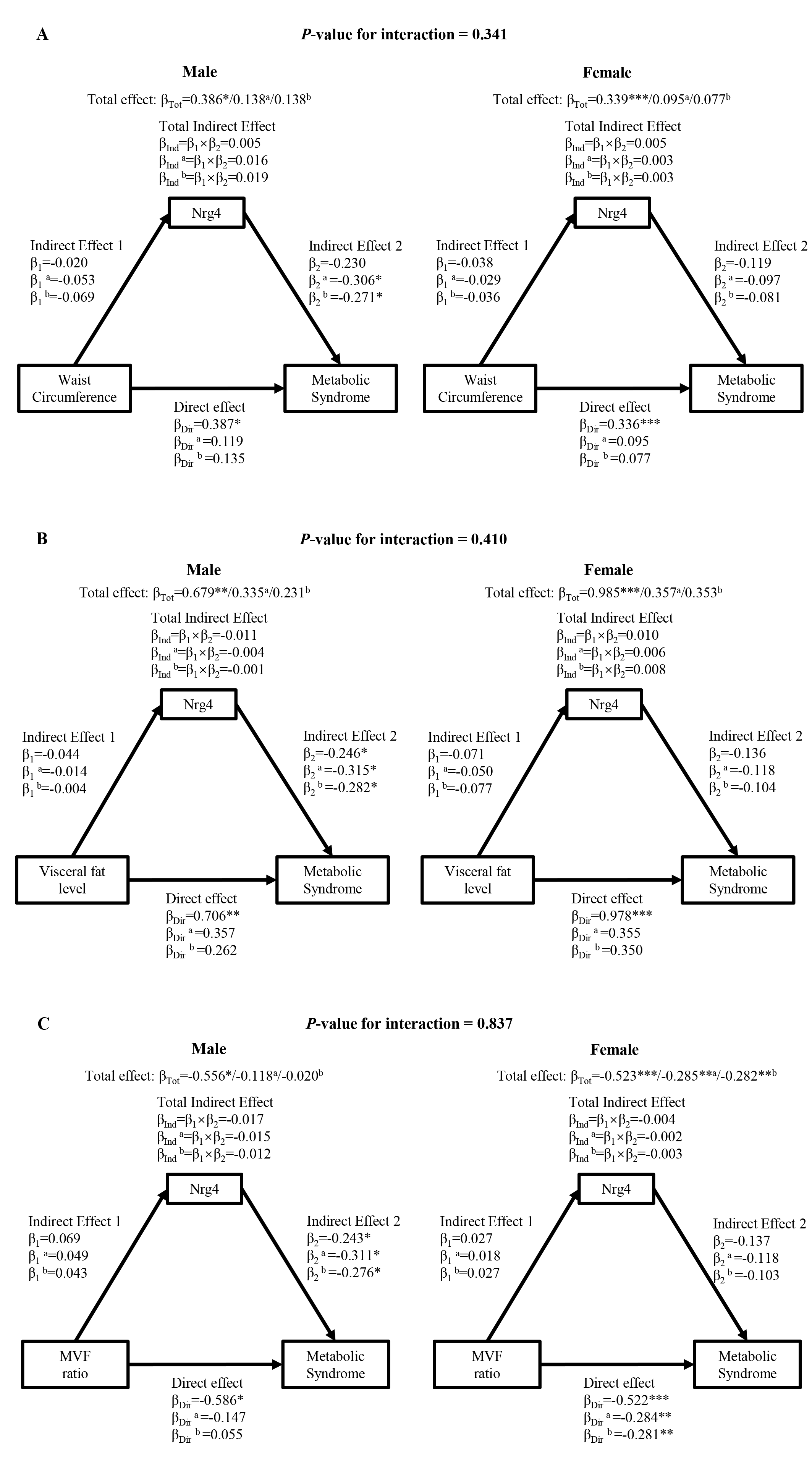

Supplement: Supplementary file 3 [file Image_3.TIF]

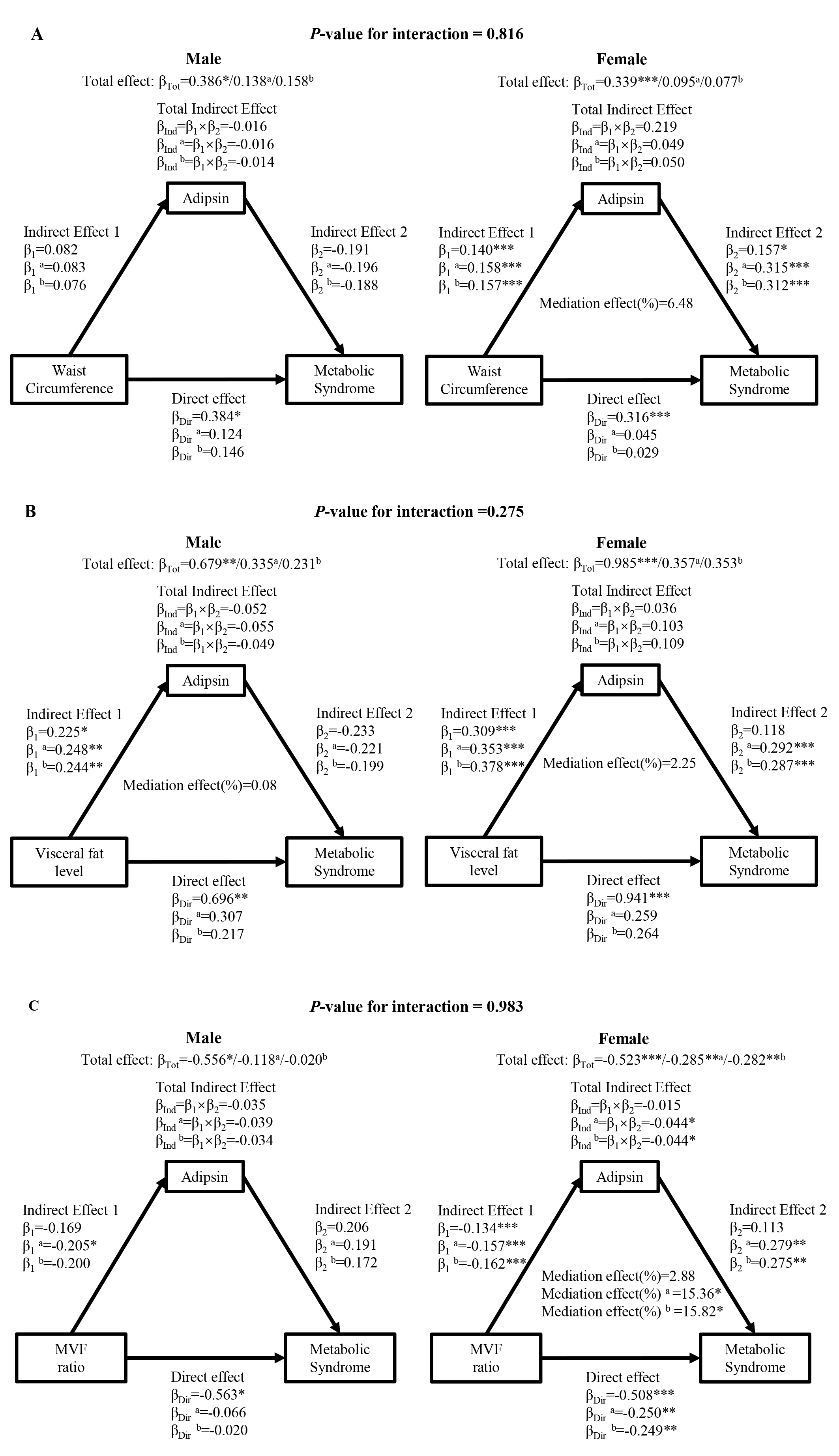

Supplement: Supplementary file 4 [file Image_4.TIF]
