# Supplementary material for: Adiposity Measurements and Metabolic Syndrome Are Linked Through Circulating Neuregulin 4 and Adipsin Levels in Obese Adults
Source: Front Physiol. 2021 May 4;12:667330. doi: 10.3389/fphys.2021.667330 (PMC8129583; doi:10.3389/fphys.2021.667330)
Supplement: Supplementary file 5 [file Table_1.DOCX]

Supplementary Table 1 Characteristics of obese subjects according to gender and metabolic syndrome.

|  | Total | | |  | Females | | |  | Males | | |
| --- | --- | --- | --- | --- | --- | --- | --- | --- | --- | --- | --- |
|  | Metabolic syndrome | Non-metabolic syndrome | P-value | Metabolic syndrome | | Non-metabolic syndrome | P-value |  | Metabolic syndrome | Non-metabolic syndrome | P-value |
| Sample size | 781 | 431 |  | 530 | | 335 |  |  | 251 | 96 |  |
| Age (years) | 54.3±7.2 | 51.6±7.2 | <0.001 |  | 54.7±6.8 | 51.3±7.2 | <0.001 |  | 53.4±7.8 | 52.9±7.2 | 0.595 |
| BMI (kg/m^2^) | 27.8±3.2 | 26.8±2.8 | <0.001 |  | 27.7±3.3 | 26.7±2.9 | <0.001 |  | 28.2±3.0 | 27.1±2.3 | <0.001 |
| Waist circumference (cm) | 94.7±7.4 | 92.2±6.6 | <0.001 |  | 93.0±7.2 | 90.9±6.6 | <0.001 |  | 98.2±6.7 | 96.5±4.9 | 0.021 |
| Current smokers (n, %) | 104(13.3) | 54(12.5) | 0.697 |  | 3(0.6) | 5(1.5) | 0.166 |  | 101(40.2) | 49(51.0) | 0.069 |
| Systolic BP (mmHg) | 139.2±16.4 | 122.6±14.2 | <0.001 |  | 137.7±16.8 | 121.6±14.8 | <0.001 |  | 142.4±15.2 | 126.2±11.1 | <0.001 |
| Diastolic BP (mmHg) | 82.3±10.3 | 73.7±8.7 | <0.001 |  | 80.8±10.3 | 73.1±9.0 | <0.001 |  | 85.6±9.7 | 76.0±7.5 | <0.001 |
| Fasting glucose (mmol/L) | 6.49±1.91 | 5.49±0.87 | <0.001 |  | 6.44±1.88 | 5.50±0.94 | <0.001 |  | 6.60±1.95 | 5.46±0.52 | <0.001 |
| 2-h glucose (mmol/L) | 9.91±4.38 | 7.23±2.28 | <0.001 |  | 9.97±4.26 | 7.33±2.26 | <0.001 |  | 9.77±4.63 | 6.89±2.33 | <0.001 |
| Fasting insulin (mU/L) | 14.86±13.64 | 10.05±4.64 | <0.001 |  | 15.41±15.47 | 10.13±4.68 | <0.001 |  | 13.71±8.49 | 9.77±4.51 | <0.001 |
| 2-h insulin (mU/L) | 94.43±71.21 | 61.70±46.69 | <0.001 |  | 98.47±71.71 | 63.06±47.48 | <0.001 |  | 85.90±69.53 | 56.94±43.75 | <0.001 |
| HOMA-IR | 3.47(2.54-4.89) | 2.19(1.56-2.99) | <0.001 |  | 3.52(2.56-4.91) | 2.21(1.57-3.00) | <0.001 |  | 3.24(2.42-4.83) | 2.14(1.52-2.85) | <0.001 |
| Triglyceride (mmol/L) | 1.97(1.37-2.67) | 1.13(0.85-1.41) | <0.001 |  | 1.88(1.29-2.53) | 1.07(0.83-1.37) | <0.001 |  | 2.21(1.65-3.20) | 1.27(1.00-1.55) | <0.001 |
| Total cholesterol (mmol/L) | 6.01±1.13 | 5.70±0.97 | <0.001 |  | 6.02±1.15 | 5.73±0.98 | <0.001 |  | 5.98±1.10 | 5.60±0.93 | 0.003 |
| LDL-c (mmol/L) | 3.68±1.04 | 3.65±0.90 | 0.533 |  | 3.72±1.04 | 3.65±0.90 | 0.319 |  | 3.61±1.06 | 3.64±0.91 | 0.834 |
| HDL-c (mmol/L) | 1.29±0.27 | 1.51±0.28 | <0.001 |  | 1.34±0.26 | 1.57±0.26 | <0.001 |  | 1.18±0.25 | 1.30±0.24 | <0.001 |
| Adipsin (μg/ml) | 5.02(4.05-6.34) | 4.84(3.93-6.16) | 0.019 |  | 4.90(3.98-6.15) | 4.68(3.81-5.90) | 0.025 |  | 5.18(4.35-6.48) | 5.64(4.49-7.07) | 0.198 |
| Nrg4 (ng/ml) | 3.24(2.40-4.52) | 3.55(2.60-5.29) | <0.001 |  | 3.47(2.62-4.73) | 3.67(2.62-5.49) | 0.003 |  | 2.90(2.02-3.86) | 3.20(2.38-4.40) | 0.050 |
| Visceral fat level | 10(8-14) | 8(7-11) | <0.001 |  | 8(8-10) | 8(7-9) | <0.001 |  | 15(14-17) | 15(14-16) | 0.003 |
| MVF ratio | 4.22±0.80 | 4.64±0.93 | <0.001 |  | 4.55±0.72 | 4.92±0.85 | <0.001 |  | 3.55±0.45 | 3.68±0.38 | 0.021 |

Data are presented as the mean± SD or median (interquartile range).

BMI = body mass index; BP = blood pressure; HOMA-IR = homeostasis model assessment of insulin resistance; LDL-c = low-density lipoprotein cholesterol; HDL-c = high -density lipoprotein cholesterol; MVF= muscle mass to visceral fat; Nrg4=neuregulin 4.

**Supplementary Figure 1. Mediation analysis model.**

**Supplementary Figure 2. Risk of the components of metabolic syndrome associated with adiposity measurements stratified by gender.**

**MVF = muscle mass to visceral fat; OR = odds ratio; CI = confidence interval.**

**Supplementary Figure 3. The mediation effect of circulating Nrg4 levels on the association between adiposity measurements and metabolic syndrome by gender.**

Panel A. Mediation analysis of Nrg4 on the association between waist circumference and MetS.

Panel B. Mediation analysis of Nrg4 on the association between visceral fat level and MetS.

Panel C. Mediation analysis of Nrg4 on the association between MVF ratio and MetS.

β=standardized regression coefficient; β_1_=indirect effect 1; β_2_ = indirect effect 2; β_Ind_=total indirect effect; β_Dir_=direct effect; β_Tot_=total effect; MVF=muscle mass to visceral fat; Nrg4=neuregulin 4.

**P* < 0.05; ***P* < 0.01; ****P* < 0.001.

Model a: adjusted for fasting insulin, 2-h glucose.

Model b: adjusted for model a + HOMA-IR.

**Supplementary Figure 4. The mediation effect of circulating adipsin levels on the association between adiposity measurements and metabolic syndrome by gender.**

Panel A. Mediation analysis of adipsin on the association between waist circumference and MetS.

Panel B. Mediation analysis of adipsin on the association between visceral fat level and MetS.

Panel C. Mediation analysis of adipsin on the association between MVF ratio and MetS.

β=standardized regression coefficient; β_1_=indirect effect 1; β_2_ = indirect effect 2; β_Ind_=total indirect effect; β_Dir_=direct effect; β_Tot_=total effect; MVF=muscle mass to visceral fat.

**P* < 0.05; ***P* < 0.01; ****P* < 0.001.

Model a: adjusted for fasting insulin, 2-h glucose.

Model b: adjusted for model a + HOMA-IR.
